# Development of a Novel Anti-Adhesive Vaccine Against *Pseudomonas* *aeruginosa* Targeting the C-terminal Disulfide Loop of the Pilin Protein

**DOI:** 10.22088/acadpub.BUMS.6.2.4

**Published:** 2017-05-31

**Authors:** Sobhan Faezi, Ahmad Reza Bahrmand, Mehdi Mahdavi, Seyed Davar Siadat, Iraj Nikokar, Soroush Sardari

**Affiliations:** 1 *Departments of Mycobacteriology and Pulmonary Research, Pasteur Institute of Iran, Tehran, Iran.*; 2 *Microbiology Research Center (MRC), Pasteur Institute of Iran, Tehran, Iran.*; 3 *Departments of Immunology, Pasteur Institute of Iran, Tehran, Iran.*; 4 *Laboratory of Microbiology and Immunology of Infectious Diseases, Faculty of paramedicine, Guilan University of Medical Sciences, Rasht, Iran.*; 5 *Biotechnology Research Center, Drug Design and Bioinformatics Group, Pasteur Institute of Iran, Tehran, Iran.*

**Keywords:** *Pseudomonas aeruginosa*, type IV pili, disulfide loop, vaccine, pET26b

## Abstract

Type IV pili (T4P) are major virulence factors of *Pseudomonas aeruginosa* (*P. aeruginosa*) that are associated with primary adhesion, biofilm formation and twitching motility. This study focuses on the introduction of a novel biologically active subunit vaccine derived from the disulfide loop (DSL) of *P. aeruginosa* pilin. We investigated the expression of the novel PilA in-frame with pET26b vector, which contains three domains, that each domain contains three tandem repeats. The flexible (GGGGS) and (GGGGS)3 linkers were linked between the three tandem repeats and each *pilA* domain, respectively. The recombinant construct (pET26b/*pilA*) was transformed and expressed in *Escherichia coli* BL21 (DE3). The reactivity of specific antiserum against PilA was assessed by ELISA method. The biological activities of this candidate vaccine were evaluated by western blotting, opsonophagocytosis and twitching inhibition assays. The pET26b/*pilA* plasmid was confirmed by enzymatic digestion. The purified PilA protein was confirmed by immunoblot analysis. The checkerboard titration showed that the optimal dilution of the antibody to react with antigen was 1:8. The results of opsonophagocytosis assay revealed that the antibodies raised against PilA promoted phagocytosis of the PAO1 and 6266E strains to some extent (17.5% and 16.3%, respectively), so the twitching inhibition test confirmed this result. Taken together, these are the preliminary results based on a first chimerical structure failure to induce antibodies that promote the opsonization and eradication of the pathogen. Therefore, the biological activity of the PilA protein showed that it should be introduced with other proteins or target antigens against *P. aeruginosa* in the future studies.

The bacterium *Pseudomonas aeruginosa* (*P. aeruginosa*) is a ubiquitous organism that is also known as an opportunistic human pathogen, causing both chronic and acute infections in susceptible populations, including individuals with burn wounds or cystic fibrosis, or intensive care unit patients (1). *P. aeruginosa* can infect almost any part of the human body, but typically targets surface-exposed epithelial cells, such as in the skin, airways and eyes. The most important question that still remains unanswered for non-obligate pathogens like *P. aeruginosa *is how these bacteria initiate infections after entering the host. Given that *P. aeruginosa* is among the many bacteria that grow as a biofilm during infection, there is a need to understand how this bacterium attaches to individual cells and form biofilm following surface colonization.

The pathogenesis of *P. aeruginosa* infections is multifactorial and includes a complex of virulence factors; hence, it has made vaccine development difficult. Bacterial attachment is an initial and a critical step for the establishment of infection that involves bacterial adhesion and host receptors. One of the most important adhesins in *P. aeruginosa* is pili (2). Type IV pili (T4P) are thin, long, flexible, and retractable protein filaments. T4P are polarly localized; filamentous surface appendages present at the cell surface of a broad range of pathogenic and environmental bacterial species (3). T4P have an average length of 2.5 μm and is 5.2 nm in outer diameter and 1.2 nm in central channel diameter (4). This adhesive cell-surface structures are the prominent virulence factors that are essential for the initiation of infection by mediating attachment to host cells, whereas non-piliated strains were reported to show a 90% decrease in their ability to bind human pneumocytes (5), and also mutant strains that are unable to produce T4P are attenuated in virulence (6, 7). Furthermore, another study revealed that non-piliated strains caused 28%–96% fewer cases of *P. aeruginosa *pneumonia as compared to piliated strains in a mouse model of infection (8). T4P plays an important role in many processes, including attachment to biotic and abiotic surfaces, DNA uptake, biofilm formation, phage transduction and a special form of bacterial cell movement, known as 'twitching motility' (9), hence, they have been used as an ideal target antigen for vaccine development.

T4P have been classified into two different subtypes, type IVa pilus (T4aP) and type IVb pilus (T4bP), based on differences in the architecture of the assembly systems and in the structure of the major pilin subunit. T4aP is found in a wide variety of bacterial species such as *P. aeruginosa*, whereas T4bP is found predominantly in enteric pathogens (3). The pilus fiber is composed of thousands of copies of PilA (or pilin, the major structural subunit) that are encoded by an operon that is positively controlled by the *algR *regulator (10). The PilA monomer can be divided into three regions: a highly conserved hydrophobic N-terminal α-helix region; a hypervariable central region, and a semi-conserved C-terminal region containing β-strands. Pili binds buccal, tracheal and corneal epithelial cells, specifically through interaction with a disulfide-loop (DSL) region that is found at the C-terminus of the pilin monomer called the receptor-binding domain (RBD). This domain of *P. aeruginosa* pilin is a suitable candidate for a peptide vaccine (4). The DSL region is structurally highly conserved among type IV pilins of all species of *P. aeruginosa*, although the size of the loop (from 12 to 31 amino acids in *P. aeruginosa*) and its sequence are varied among pilin alleles. The C-terminal DSL of the pilin subunit was shown by monoclonal antibodies that mediate attachment to epithelial cell receptors, this finding suggests that PilA itself acts as both a major structural subunit and an adhesin (7, 11). There are several studies mentioning that the attachment of *P. aeruginosa* to the respiratory mucosa, skin, corneal surfaces is multifactorial. *P. aeruginosa* type IV pili are thought to interact with the glycosphingolipid epithelial receptor asialo-GM1 (aGM1) via the DSL, which is proposed to be exposed only at the tip of pilus. The membrane distribution of asialo-GM1 is compared to that of caveolin-1 and understanding lipid partitioning between membrane domains (11, 12). Finally, T4P from all strains of *P. aeruginosa* shares a common receptor. However, the sequence diversity presents a significant obstacle to the development of a broadly protective RBD-based vaccine targeting the T4P. 

The pET-based vectors are the most powerful systems, yet developed for the cloning and expression of recombinant proteins in the *E. coli*. One vector of this system, pET26b(+) (abbreviated as pET26b), was used in this study for the construction and expression of recombinant proteins. This expression vector is under the control of the T7 promoter and carries an N-terminal PelB signal sequence for potential periplasmic localization of recombinant protein and optional C-terminal His-tag sequence for detection and purification (13). In the present study, we designed a chimeric plasmid that contains *pilA* region, which encodes the three peptides of DSL (in triplicate forms). The *pilA* region contains three domains, *pilA* 1, *pilA* 2 and *pilA* 3, in which each domain comprises three copies of the gene. The* pilA* 1 domain which includes three tandemly repeated copies encodes a 17-residue sequence of PilA 1 peptide (4). The* pilA* 2 and *pilA* 3 domains have also contained three tandemly repeated copies of the 19-residue sequence that encoded the PilA 2 and PilA 3 peptides, respectively (manuscript under preparation). We used L1 and L2 as a flexible, controllable and soluble linkers between tandem repeats and domains, respectively (see Table1).

Therefore, we have designed a novel recombinant construct containing nine tandem copies of the DSL sequence in concert with pET26b vector and then purified the recombinant PilA (r-PilA) protein as a possible vaccine candidate against *P. aeruginosa* specially in the lung infections. For the first time, we report the purification, characterization and functional activity of a novel chimeric PilA protein from *P. aeruginosa*.

## Materials and methods

**Bacterial strains, plasmid, and media**

*Escherichia coli* (*E. coli*) strains BL21 (DE3) and Top10, as expression and preservation hosts, were preserved in our laboratory. The *P. aeruginosa* laboratory strains PAO1 and 6266E (a clinical piliated strain that was kindly obtained by Shahid Beheshti University of Medical Sciences, Tehran, Iran) were used (Table 2). The recombinant plasmid pET26b/*pilA* (recombinant secretory expression vector) was synthesized by Biomatik Corporation (Cambridge, Ont., Canada). All enzymes for DNA manipulations were obtained from NEB (USA). The anti-His (C-Term)-HRP monoclonal antibody was obtained from Invitrogen (USA). Ni^2+^-NTA agarose was purchased from Qiagen (USA). The strains were cultured in Luria-Bertani (LB) broth or on agar (Merck, Germany) at 37 °C with or without 30 µg kanamycin/ml (Bioscience, Canada).

**Table 1:** The oligonucleotide and amino acid sequences that used to design the tandem pilA region on the pET26b plasmid**.**

**Domains and linkers**	**Nucleotide sequence**	**Amino acid sequence **	**References**
*pilA* 1	GCGTGCAAAAGCACCCAGGACCCGATGTTTACCCCGAAAGGTTGCGATAAT	ACKSTQDPMFTPKGCDN	4
*pilA* 2	TGCAACATAACCAAAACGCCGACTGCGTGGAAACCGAACTATGCGCCAGCTAACTGT	**C**N**I**TKT**P**TA**WK**P**NYAPANC**	
*pilA* 3	TGCGCTATTTCAGGAAGCCCGGCAAATTGGAAGGCGAATTATGCTCCGGCGAATTGT	**C**A**I**SGS**P**AN**WK**A**NYAPANC**	*
L1	GGGGGCGGGGGCTCC	GGGGS	this study
L2	GGGGGCGGGGGCTCCGGGGGCGGGGGCTCCGGGGGCGGGGGCTCCGGGGGCGGGGGCTCC	(GGGGS)_3_	this study

Bold characters in amino acid sequence of pilA 2 and pilA 3 represent conserved and other represent semi-conserved area.

* manuscript under preparation

**Table 2:** The characteristics of the studied strains.

**Host**	**Relevant characteristics**
*E. coli* BL21 (DE3)	Host strain for overexpression of the recombinant protein from the T7 promoter
*E. coli* Top10	Host strain for proliferation of the recombinant vector
*Pseudomonas aeruginosa* PAO1	common reference strain in laboratory, spontaneous chloramphenicol-resistant mutant, non-mucoid strain
*Pseudomonas aeruginosa* 6266E	A clinical isolated from cystic fibrosis patient, hyper mucoid strain

**Construction of the expression vector**

The *pilA* gene was inserted into the *E. coli* expression vector pET26b, in the frame with the PelB signal peptide, a T7 promoter, kanamycin resistance gene and the C-terminal six-His tagged sequence. The *pilA* region flanked by a BamHI at the 3′ end and an XhoI at the 5′ end of the encoding region. The tandem *pilA* region contains three domains, *pilA* 1-3, that each domain contains three tandem repeats. The glycine/serine as a flexible linker was used between the three monomer sequences of *pilA* (GGGGS, called L1) and between each domain ((GGGGS)_3_, called L2). The flexible linkers comprised glycine and serine, were used to avoid the interference resulting from the direct ligation between epitopes or subsequent generation of new epitopes. As shown in Table 1, the *pilA* 1 includes three copies of 17-amino acid residues, while the *pilA* 2 and 3 domains contain three copies of 19-amino acid residues. In designation of the construct, we inserted a nucleotide G before the start codon ATG immediately after the BamHI site (ggatcc**G**ATG) of the pET26b vector, resulting in correct framing of the inserted gene.

After the plasmid was transformed into *E. coli* Top10 competent cells, transformants were selected on LB plates (1% tryptone, 0.5% NaCl, 0.5% yeast extract, 1.5% agar, pH 7.5) supplemented with 30 µg/ml kanamycin. The recombinant plasmid pET26b/*pilA* was verified by restriction enzyme digestion. The vector was treated with the restric-tion endonucleases BamHI and XhoI (Jena Bio-science Kit, Germany) according to manufacturer’s instruction. The digested fragments were separated by 1.2% (w/v) agarose gel electrophoresis. 

**Expression and isolation of the recombinant protein**

The expression and purification of the r-PilA protein was performed as previously described (14), with slight modification. The* E. coli* BL21 (DE3) was transformed with the plasmid pET26b/*pilA*. A single colony was grown overnight in 5 ml LB medium supplemented with kanamycin (30 µg/ml) and then inoculated into fresh one liter LB medium containing kanamycin using 1% of the final growth volume. When culture was grown to logarithmic growth phase (~ OD_600_ of 0.8), IPTG was added at different concentrations (final concentration of 0.1, 0.5, 1 and 10 mM) and at different time intervals (1 to 18 h). The cells were harvested at 8000 g for 30 min (7 g of wet weight cell pellet), and the periplasmic *E. coli* fraction was extracted via osmotic shock procedure. The harvested cells were suspended in 25 ml hypertonic solution (30 mM Tris, 20% w/v sucrose, 0.5 mM ETDA, pH 8) and incubated for 30 min at 4 ºC. Cells were pelleted, and the supernatant was collected. The cells were re-suspended in 25 ml hypotonic solution (5 mM MgSO_4_) and incubated for 30 min at 4 ºC followed by an additional centrifugation. The supernatants from the hypotonic solution and hypertonic solution were combined, centrifuged to remove debris, dialyzed against phosphate-buffered saline (PBS, pH 7.4) and finally clarified over a 0.45 µm filter. The clarified osmotic shock fluid was loaded onto the pre-equilibrated HisTrap affinity column and washed with equilibration buffer (20 mM Tris, 300 mM NaCl, 40 mM imidazole, pH 8) for six column volumes. Bound proteins were eluted with three column volumes of 20 mM Tris, 300 mM NaCl, 500 mM imidazole, pH 8. Fractions with an absorbance at 280 nm greater than 0.05 were pooled, and buffer exchanged into PBS, and subjected to analysis by 12% SDS–PAGE. The concentration of purified r-PilA protein was quantitatively measured using a NanoDrop 2000c (Thermo Scientific, USA) and Bradford protein assay using standard albumin (Sigma, USA) and finally aliquoted in 0.5 mg/ml vials.

**Immunoblot analysis**

The purified r-PilA was electrophoresed in a 12% SDS–PAGE and then transferred onto PDVF membrane (Hi-bond Amersham Biosciences, USA) using a Mini-PROTEIN tetra cell (Bio-Rad, USA) at 100 mA for 1 h. The membrane was then blocked for 60 min in 5% (w/v) skim milk (Merck, Germany). After blocking, the membrane was transferred to a tray containing the anti-His (C-term)-HRP antibody (Invitrogen, USA) diluted 1:5000 in blocking buffer and then incubated for 2 h with gentle agitation. The membrane was then washed 5 times in TBS-T (Tris buffer saline contains 0.1% Tween-20) for 5 min each. Finally, it was developed by the addition of 3, 3′-diaminobenzidine (DAB) (Sigma, USA) solution containing 0.1% hydrogen peroxide allowing it to incubate until bands were seen. The reaction was stopped by rinsing with water.

**Mice immunization and serum preparation**

Five to seven-week-old female BALB/c mice were obtained from Pasteur Institute of Iran (Karaj, Iran). Mice were housed for one week before the experiment, had free access to food and water and maintained in a light/dark cycle with lights (12 h/12 h). All experiments were in accordance with the Animal Care and Use Protocol of the Pasteur Institute of Iran. These inbred mice were assigned into a group containing four mice. The mice were immunized (5 µg per nostril) intranasally with r-PilA protein at zero time. Immunized mice were boosted twice in 2-week intervals. Prior to the first immunization and 2 weeks after each immunization, the blood was collected from the orbital sinus and then sera were taken by centrifugation. 

**Enzyme-linked immunosorbent assay (ELISA)**

To detect the humoral immune responses against vaccine candidate, an optimized indirect ELISA was performed. Briefly, 96-well immunoassay plates (Nunc MaxiSorp) were coated with 0.5 μg of the r-PilA in 100 mM sodium carbonate/sodium bicarbonate buffer (pH 9.6) and incubated overnight at 4 ºC. The plate was then rinsed three times with PBS-T (140 mM NaCl, 2.7 mM KCl, 10 mM Na_2_HPO_4_, 1.8 mM KH_2_PO_4_, 0.05% Tween 20) and incubated with 5% skimmed milk in PBS-T for 2 h at 37 ºC. After three times washing with PBS-T, a serial dilution (from 1:4 to 1:128) of mouse serum in blocking buffer was prepared and 100 µl of each one was added to each well. Following 2 h incubation at 37 ºC, plates were rinsed three times with PBS-T, and 1:10000-diluted HRP-labeled sheep anti-mouse IgG was added to each well and incubated for 1 h at 37 ºC. After washing again five times washings, the enzymatic activity was determined by adding 100 µl of 3, 3, 5, 5’-tetramethyl benzidine (TMB) as substrate solution. Following incubation for 30 min, the reaction was stopped with adding 100 µl of 2N H_2_SO_4_ and the optical density was read at 450 nm with an ELISA plate reader (Awareness Stat Fax 2100, USA).

**Opsonophagocytic killing assay (OPA)**

The ability of antisera to opsonize *P. aeruginosa* for killing in the presence of complement and macrophage was evaluated using an *in vitro* OPA as described previously (15). Briefly, four different dilutions (1:4, 1:8, 1:16 and 1:32) of anti r-PilA antibody were used as an opsonin. Complement activity of antisera was removed by heating at 56 ºC for 30 min. The infant rabbit serum (Pasture Institute of Iran, Karaj, Iran) was used as a complement source. For the opsonophagocytic assay, the bacteria (2×10^9^ cells per well) were first incubated with an equal volume of diluted and heat-inactivated polyclonal IgG at 22 ºC for 60 min. For the elimination of excessive antibodies, they were washed twice with BSA (1% (w/v)). After suspending with 200 µl of 1% BSA, 100 µl of mouse macrophages (2×10^7^ CFUs/ml) was mixed with 100 µl complement in sterile 48-well microfuge plate (Greiner bio-one, Germany), and then incubated in a shaker at 37 ºC for 90 min. After 90 min incubation, 25 µl of the mixture was diluted in 225 µl saline and finally plated for bacterial enumeration. Non-immune mouse serum (NMS) (1:4 dilution) was used as pre-immune serum (control IgG). Control reactions excluded complement and/or antibody, or used pre-immunization serum. This assay was performed in duplicate for each quantity. The opsonic killing activity of immune sera was compared to that of sera obtained before vaccination (pre-immune sera). The opsonophagocytosis activity of the serum was calculated as follows;

Percentage of opsonophagocytosis= (1-(CFU of immune serum at 90 min / CFU of pre-immune serum at 90 min))×100

**Twitching inhibition test**

To verify the functionality of the specific polyclonal antibody, the twitching inhibition assay was carried out according to Castric et al.(16) as follows. A 1:8 dilution of specific polyclonal anti r-PilA antibody (filter-sterilized) was added to LB broth (containing 1% agar), which was poured into a 15×90 mm plastic Petri dish. After solidification, the plate was dried at room temperature for 6 h. Single colonies of the *P. aeruginosa* PAO1 and 6266E strains to be tested were stab-inoculated with a toothpick to the bottom of the plates. After 18 h incubation at 37 ºC, the diameter zone of growth of different strains obtained at the interstitial surface of the agar and the plate was measured. For each assay, triplicate plates were examined.

**Statistical analysis**

Data were analyzed by ANOVA or Kruskal–Wallis test, depending on the assay. Differences were considered significant at p less than 0.05. Statistical analysis was performed using the software GraphPad Prism version 6.0 for Windows, (GraphPad Software, San Diego, CA, USA).

## Results

**Construction of plasmid for periplasmic purification**

**Fig 1:**
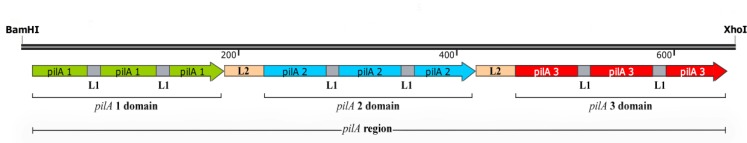
Schematic representation of the desired *pilA* region

**Fig. 2:**
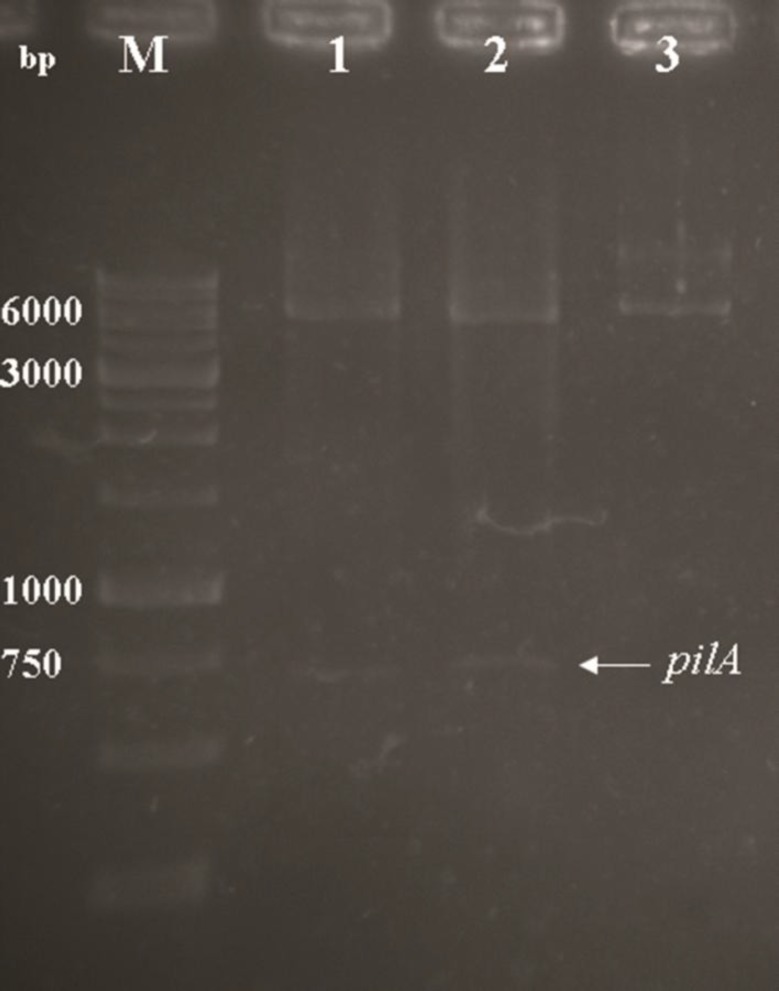
Agarose gel electrophoresis of recombinant pET26b/*pilA* with restriction enzyme digestion. (Lane M); DNA marker (1 kb), (Lane 1 and 2); BamHI/XhoI double digested recombinant vector with BamHI and XhoI buffers, respectively. Two expected fragments were observed on the gel (≈ 649 and 5360 bp bands). (Lane 3); BamHI mono digested of pET26b/*pilA* plasmid (white arrow, ≈ 6009 bp

To overcome the problems associated with cytoplasmic expression of the r-PilA protein, the plasmid pET26b was constructed for periplasmic expression of the protein. The coding sequence of tandem repeats of three *pilA* domains with specific linkers, was constructed in the pET26b expression vector (Table 1). The BamHI and XhoI restriction sites were introduced upstream and downstream of the tandem *pilA* region, respectively. Therefore, the coding sequence was preceded by a pelB signal sequence at the N-terminal region and a six His-tag at the C-terminus of the gene. On the analogy of this, the construction of the *pilA* region has been shown in Figure 1. The recombinant construct was transformed into *E. coli* Top10 cells and selected on LB containing kanamycin (30 µg/ml). Transformants were characterized by enzymatic digestion. The recombinant plasmid, pET26b/*pilA*, was extracted and its orientation confirmed by digestion with two restriction enzymes was mentioned above. The two expected bands were observed on gel: 649 and 5360 bp bands (Fig. 2). Sequence analysis of recombinant pET26b/*pilA* confirmed that there were no amplification errors and that construction was accurate.

**Fig. 3:**
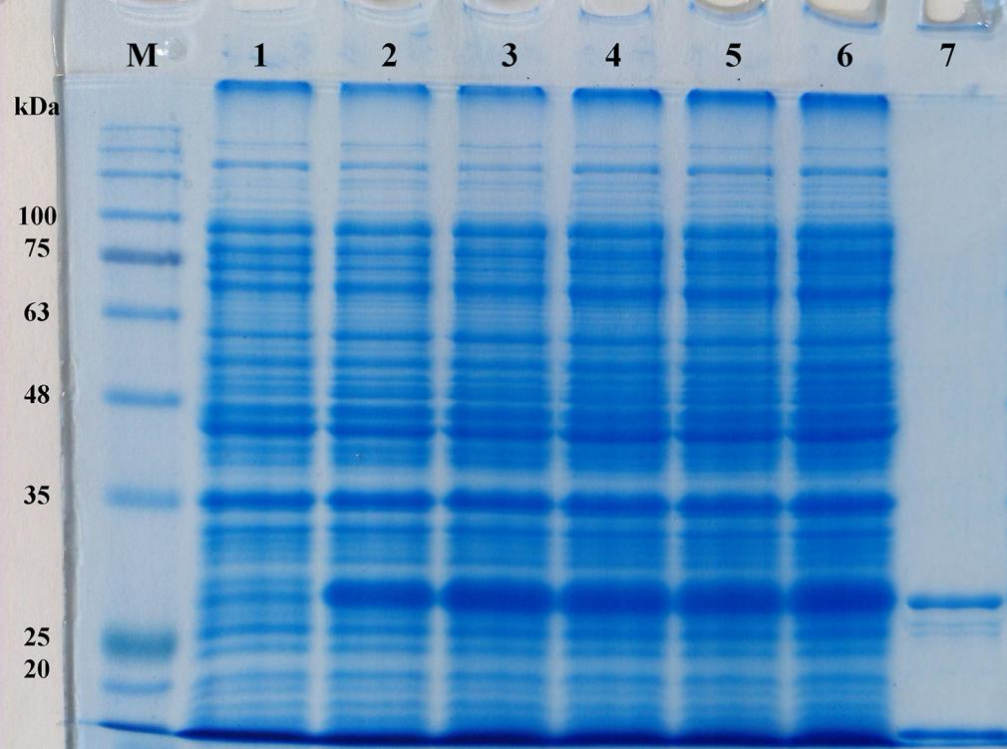
SDS-PAGE analysis of the expression of r-PilA protein in *E. coli*. The total proteins of the *E. coli* BL21harboring pET26b/*pilA* plasmid was harvested and loaded on 12% (v/v) SDS-PAGE after for 5 h induction with or without IPTG. (Lane M) denote molecular weight marker proteins; (lane 1) total cell lysate of non-induced bacteria; (lanes 2-6) 1-5 hrs after induction with IPTG, respectively; (lane 7) purified r-PilA protein (≈ 28 kDa) after HiTrap Chelating and Ni^2+^-affinity chromatography

**Fig. 4:**
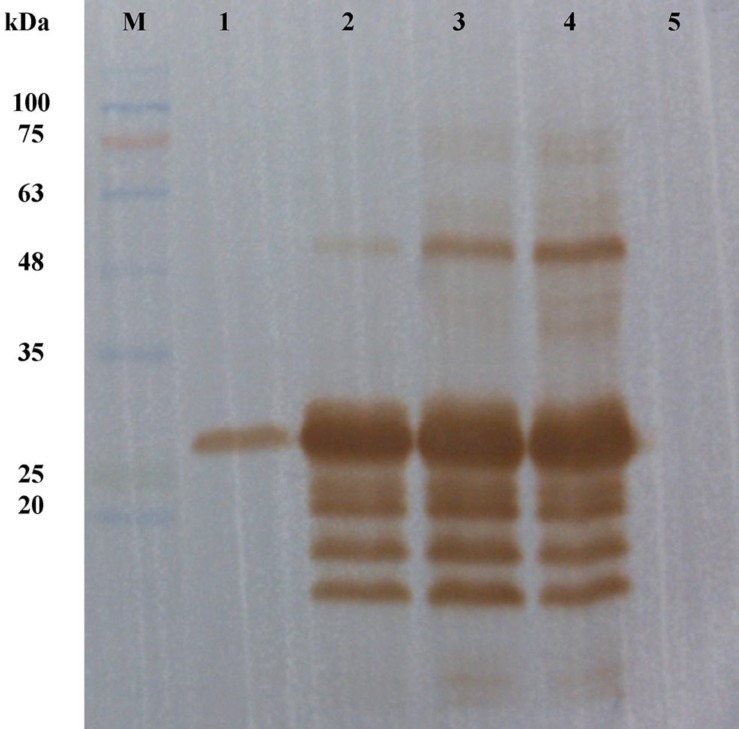
Western blot analysis of the expressed r-PilA-6His tag protein in *E. coli* BL21. After running the SDS-PAGE, the protein transferred onto PVDF membrane and detected with an anti-His monoclonal antibody. (Lane M) PageRuler^TM^ Prestained Protein Ladder; (lane 1) purified periplasmic r-PilA-6His by Ni^2+^-NTA agarose via osmotic shock procedure; (lane 2-4) total cell lysate of induced bacteria after 3 h induction; (lane 5) total cell lysate of non-induced bacteria

**Fig. 5.**
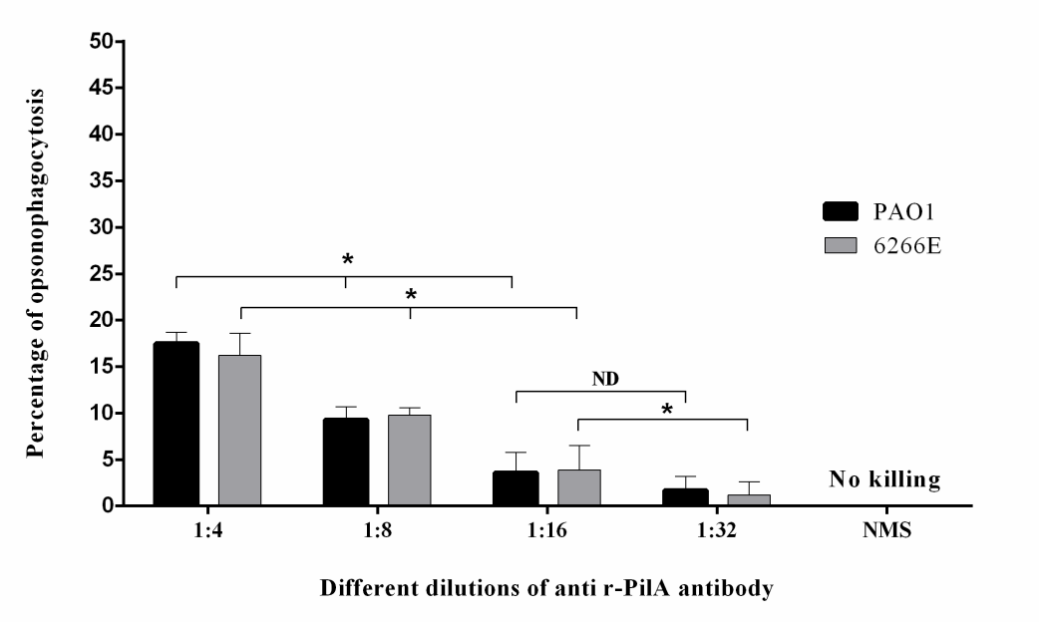
The opsonic killing activity of four different dilution of anti r-PilA antibody against *P. aeruginosa* strains PAO1and 6266E. To do this test, desired strains were incubated with different dilutions of mouse anti r-PilA antiserum and mouse macrophage in the presence of rabbit complement. The opsonic killing activity was observed when specific r-PilA antibody was treated with PAO1 and 6266E strains. No cross reaction was detected between NMS and the strains. Bars represent means of triplicate determinations, and error bar indicate SD. Results were accepted to be significant at *P* less than 0.05. The Asterisks represents the groups which were significantly different (*P* < 0.05) and ND represents non-detectable difference

**Fig 6:**
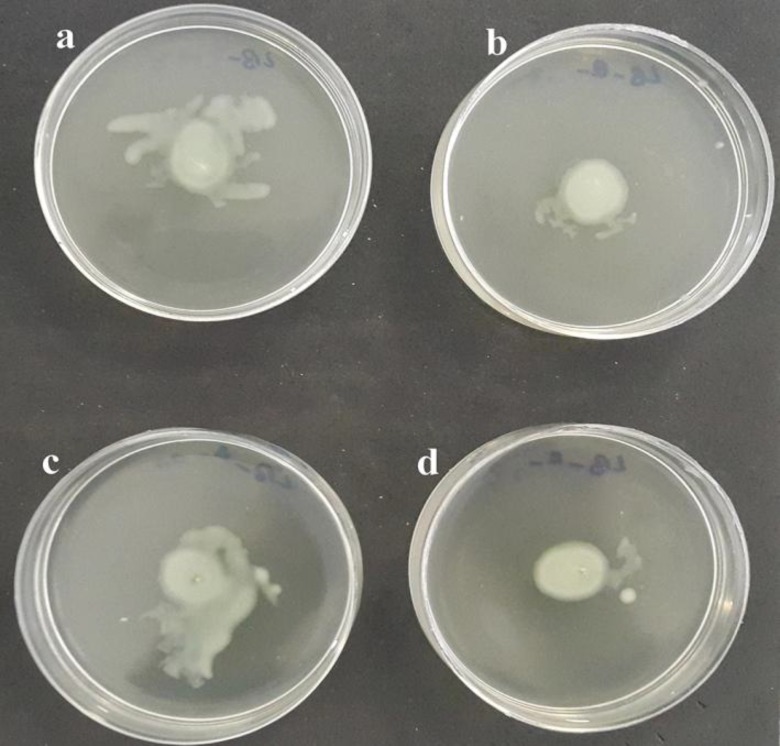
Twitching inhibition of *P. aeruginosa* PA01 and 6266E strains in the presence of r-PilA antibody on motility agar (LB broth with 1% w/v agar). The motility agar plates supplemented with a 1:8 dilution of anti r-PilA antiserum or NMS were stab inoculated with *P. aeruginosa* and incubated for 18 h at 37 °C. The antiserum raised against r-PilA inhibited motility of *P. aeruginosa* strains PAO1 and 6266E (b and d, respectively) compared with NMS (a and c, respectively

**pression and confirmation of the r-PilA**

To construct an over-expression system, the coding sequence of the *pilA *gene, whose theoretical molecular weight is approximately 25 kDa, was constructed into the expression vector pET26b to express a C-terminal His-tagged recombinant protein under the control of the strong T7 promoter in *E. coli*. The recombinant plasmid was transformed into *E. coli* BL21 (DE3). This *E. coli* strain carries a chromosomal copy of the T7 RNA polymerase gene under the control of the lacUV5 promoter. The addition of IPTG induces expression of T7 RNA polymerase resulting in transcription of the target gene under the control of the T7 promoter in cells harboring the pET26b/*pilA* vector. The N-terminal pelB secretion signal targets the translated protein in its unfolded state to the *E. coli* periplasm via the Sec-dependent transport pathway. The signal peptide is cleaved and the protein folds in the periplasm with the help of chaperones and isomerases. Results from SDS-PAGE analysis of expression products showed that the optimum protein expression was 2 h after induction with IPTG at the final concentration of 1 mM. The expression product of the r-PilA was approximately 28 kDa in molecular weight (Fig. 3). Furthermore, almost all the protein was present as soluble protein in the cell disruption supernatant. Western blot analysis on total cell extracts (induced and non-induced) and purified protein using anti His-tag-specific antibodies showed that the protein was substantially expressed using the pET26b/*pilA* expression vector when IPTG was added at the early-exponential phase of growth and after collecting the cells 2 h after induction (Fig. 4). The checkerboard titration demonstrated that the optimal dilution of the anti r-PilA antibody to react with the r-PilA was 1:8. The yield of purified fusion protein was about 2.77 mg per liter of culture media.

**Opsonophagocytic killing activity**

We next performed an opsonophagocytic assay to test whether the r-PilA-specific antibodies present in the immunized mice sera could mediate killing of *P. aeruginosa* PAO1 and 6266E strains by macrophages in the presence of complement. Unlike the NMS, the sera from mice immunized with the r-PilA showed opsonophagocytic killing activity against PAO1 and 6266E strains, to some extent, so that the number of viable bacterial cells decreased over 17.5% and 16.3% respectively, after 90 min compared with the control group (Fig. 5). In the presence of NMS, as the control group, no opsonic activity was observed. These data indicate that the anti r-PilA antibodies have a partial opsonic activity for killing of the *P. aeruginosa* strains. 

**Twitching inhibition assay**

Immunized and non-immunized mouse sera were tested in the twitching inhibition assay to evaluate their functional activity for immobilization of *P. aeruginosa* strains PAO1 and 6266E. In this assay, NMS was used as control. As shown in Figure 6, the r-PilA antibody was able to inhibit slightly the motility of the PAO1 strain at a dilution of 1:8. This antibody showed cross reactivity with 6266E strain as it slightly inhibited the motility of the strain. The two strains were motile in the presence of NMS.

## Discussion

*Pseudomonas aeruginosa* is an opportunistic pathogen which is commonly considered as an infectious agent in immunocompromised hosts. In these patients, infections are often severe and life-threatening. There is great interest in developing a vaccine to prevent *P. aeruginosa* infection, given that immunization is always considered the most economic and efficient means for such prevention, especially in developing countries. The selection of antigenic targets is critical in the design of a vaccine. It has been suggested that adhesins act as key roles in the initial stages of infection, where they allow the bacteria to anchor themselves to the epithelial layer, which can lead to successive colonization and potential invasion (17). A large number of published data showed that pili might be the most definitive antigen candidate (18, 19). T4P are essential virulence factors of *P. aeruginosa* that have been extensively studied in the mouse model (8, 20). Because of its length, the T4P has been associated with mediating of initial attachment of the bacteria to host surfaces before other adhesins secure the attachment. As soon as attachment occurs, the coordinated expression of other virulence factors facilitates invasion. Because of its early role in the pathogenesis of infection, the T4P has been suggested as an attractive vaccine target (21). A number of studies based on monoclonal antibody-binding data showed that the C-terminal DSL of the pilin subunit mediates attachment of bacteria to epithelial cell receptors (7, 11). In another study, it has been demonstrated that antibodies raised against the RBD can block pilus mediated adhesion and are the basis for this vaccine development project (22). Since adhesion and colonization play important roles in pathogenicity, as a aresult, we developed a novel DSL-based PilA protein to combat infection. cohseguently, in the current study, we produced a novel *P. aeruginosa* PilA protein by recombinant technology.

The pET system was chosen because it is a very powerful system developed especially for the cloning, expression and purification of recombinant proteins in *E. coli. *A series of vectors with signal sequence-directed secretion has been designed such as pET26b. The pET26b vector produces a recombinant protein with signal peptide PelB at the N-terminus for periplasmic localization and a His-tag at the C-terminus for detection and purification. Our major focus was the efficient translocation of the PilA protein into the periplasmic space and isolation of His-tagged proteins using the osmotic shock process. Secretion of the protein into the periplasmic space of *E. coli *may be an efficient means of obtaining correctly folded, active proteins.

In our previous study, we investigated the sequence diversity of *pilA* gene among CF isolates obtained from Tehran hospitals. After amplification of *pilA* gene by specific primers, the PCR products were sequenced. Alignment of the sequencing data showed that there are two consensus sequences with over 89% homology at the C-terminal region of the PilA protein (manuscript under preparation). As a consequence, the coding sequence of six copies of 19-amino acid residues and three copies of 17-amino acid residues (4) of the DSL region of the PilA protein was designed in the pET26b vector.

The most commonly used flexible linker has the sequence consisting of glycine and serine residues (“G/S” linker). An example of the most widely used flexible linker has the sequence of (Gly-Gly-Gly-Gly-Ser)_n_ that has been used in this study. The length of this G/S linker can be optimized to maintain necessary inter-domain interactions, or to achieve appropriate separation of the functional domains (23, 24). Flexible G/S linkers have been utilized to improve the folding and function of epitope-tagged proteins. It has also been demonstrated that the poly-G linkers provided maximum conformational freedom, but failed to ensure optimal stability (23, 25).

It is indicated that different routes of immunization could affect different levels of immune response (26). In the present study, the mice immunization was performed via intranasal (i.n.) route. It has been well documented that in. delivery of vaccine has an appropriate influence on induction of mucosal (especially IgA response) and systemic immune reactions against respiratory pathogens. In addition, easy accessibility to nasal cavity, needle-free injection and low antigenic dose are some other advantages of in. immunization (27).

In this work, a protocol was examined to express and purify the recombinant PilA protein in *E. coli* expression strain BL21 (DE3). Since the PilA protein contains at least 18 cysteine residues, it must be expressed as a secretory strategy; hence we constructed the *pilA* gene into the pET26b vector. If the protein is expressed (for example, under pET28a vector) as inclusion bodies in *E. coli*, it will contain intramolecular misfolding due to aggregation of the target protein. The promising results from the r-PilA protein purification indicate a general beneficial effect on the solubility of cysteine-containing proteins using a secretory system. Due to the ease, time effective and low cost of this purification process, we recommend this vector and strain for similar projects as well as more directed studies regarding one or few hard-to-get protein targets.

Following investigation of phagocytosis of anti r-PilA antibody on *P. aeruginosa*, we concluded that antibodies raised against r-PilA have partial opsonic killing activity (17.5% compared to control group after 90 min) following treatment with PAO1 strain. This antiserum induced phagocytosis of 6266E strain, to some extent (16.3%). These results indicate that the anti r-PilA antibodies cannot act as a suitable bioactive opsonin for complete eradication of the organism and also suggest that these antibodies probably have limited protective value. Although our findings show that the antisera to r-PilA had had not sufficient opsonophagocytic activity to support the bioactivity of our vaccine candidate, the presence of low opsonic killing activity is overall a desirable feature of the vaccines. We also showed that the antiserum raised against r-PilA can slightly inhibit the twitching motility of PAK and 6266E strains, and that the result was in agreement with the results of opsonophagocytosis.

In conclusion, due to complications arising from antigenic competition, in this study, we have provided a new strategy to develop a novel multivalent RBD-based vaccine targeting the T4P that can be used for prevention and therapeutic purposes against the pathogen. We concluded that the antibodies raised against PilA can promote (to some extent) the opsonization and eradication of the *P. aeruginosa* under *in vitro* condition. For enhancement of vaccine efficacy, it is suggested that the PilA protein is combined with surface antigenic proteins or other target antigens as an adjuvant or vaccine in the future studies.
